# Real-world safety of difelikefalin for chronic kidney disease–associated pruritus: initial insights from a European managed access programme

**DOI:** 10.1093/ckj/sfaf297

**Published:** 2025-09-24

**Authors:** Joerg Latus, Gert Mayer, Carlos Narvaez, Marius Manu, Sara Jesus, Despina Ruessmann, Lucio Manenti

**Affiliations:** General Internal Medicine and Nephrology, Robert Bosch Hospital, Stuttgart, Germany; Department of Internal Medicine IV (Nephrology and Hypertension), Medical University Innsbruck, Innsbruck, Austria; Servicio de Nefrología, Hospital Universitario Puerta del Mar, Cádiz, Spain; CSL Vifor, Switzerland; CSL Vifor, Switzerland; CSL Vifor, Switzerland; Nephrology Unit, Azienda Sanitaria Locale-Spezzino, La Spezia, Italy

**Keywords:** CKD, difelikefalin, haemodialysis, itch, safety

## Abstract

**Background:**

Chronic kidney disease–associated pruritus (CKD-aP) is a debilitating condition with limited treatment options. A managed access programme (MAP) began in October 2021 to provide early, controlled access to the novel antipruritic difelikefalin to European and Australian patients with CKD-aP with no local access to commercially available treatments. Here, we describe the safety data collected up to 31 October 2024.

**Methods:**

Eligible adults with moderate-to-severe CKD-aP receiving in-centre haemodialysis (HD) were provided with difelikefalin (0.5 µg/kg) intravenously after each HD session. All adverse events (AEs) were recorded; a Global Drug Safety team assessed seriousness and causality. AE details, including outcomes, were also recorded.

**Results:**

A total of 438 patients were provided with a median of 115 (min–max: 30–1035) days of difelikefalin treatment. Of these, 167 (38.1%) patients experienced 458 AEs. Of 246 serious AEs (SAEs), 10.2% were considered possibly related to difelikefalin. Of those with a known outcome, 88.0% were resolved during the MAP. There were 63 fatal SAEs, none considered related to difelikefalin. There were 152 non-serious AEs, of which 63.8% were deemed possibly/probably related to difelikefalin. Of the 59 difelikefalin-related non-serious AEs with a known outcome, 86.4% were resolved during the MAP. Most AEs and SAEs were consistent with conditions typical of patients with CKD requiring HD and/or the known safety profile of difelikefalin.

**Conclusions:**

No new safety signals were detected in this MAP analysis over a 3-year period. The overall safety results were consistent with the known safety profile for difelikefalin patients with moderate-to-severe CKD-aP receiving HD.

KEY LEARNING POINTS **What was known:**

- Chronic kidney disease–associated pruritus (CKD-aP) is a common condition in patients with CKD, particularly (but not exclusively) in those undergoing haemodialysis (HD); patients with CKD-aP have significantly impacted health-related quality of life and limited treatment options.
- Difelikefalin is a selective, kappa opioid receptor agonist approved by the US Food and Drug Administration and European Medicines Agency for the treatment of moderate-to-severe pruritus in patients undergoing HD.
- Managed access programmes (MAPs) provide a route for patients who may otherwise have limited access to new and novel treatments; a MAP was initiated in October 2021 to provide patients with end-stage kidney disease access to difelikefalin before it became commercially available.

**This study adds:**

- No new safety signals for difelikefalin were identified in a real-world analysis of MAP data in Europe and Australia, which provided difelikefalin to adult patients with moderate-to-severe CKD-aP.
- Most adverse events and serious adverse events observed were consistent with conditions typical of patients with CKD requiring HD and/or the known safety profile of difelikefalin; no fatal events reported were considered related to difelikefalin.
- This analysis confirms the safety profile from the Phase 3 clinical trial programme of difelikefalin in a real-world setting.

**Potential impact:**

- Given the heterogeneous nature of real-world populations and the complexity of chronic conditions such as CKD-aP, this MAP provides evidence for the safety profile of difelikefalin in a less controlled setting than in clinical trials, and may provide reassurance to clinicians regarding the safety profile of difelikefalin.
- Considering the available Phase 3 clinical efficacy data, it can therefore be concluded that difelikefalin is a favourable option for the treatment of CKD-aP in patients receiving HD.

## INTRODUCTION

Chronic kidney disease–associated pruritus (CKD-aP) is a common condition experienced by patients with chronic kidney disease (CKD), particularly, but not exclusively, in those undergoing haemodialysis (HD) [1, 2]. The condition is frequently underestimated and underreported [3–5]. Despite this, it can significantly affect health-related quality of life (HRQoL) [2, 6–9], with its impact on HRQoL likened to that of chronic pain [10]. Patients have limited treatment options and often have to rely on suboptimal management through off-label use of treatments with limited evidence of efficacy [2].

Overall, CKD-aP has a significant impact on both clinical and patient-reported outcomes and leads to considerable health challenges [1]. For example, CKD-aP-affected patients exhibit poor-quality sleep, a longer post-dialysis recovery time, and may have higher morbidity and mortality rates than patients with CKD who do not have pruritus [11–13].

Difelikefalin is a selective, kappa opioid receptor agonist [14, 15] that demonstrates anti-pruritic effects by activating kappa opioid receptors on peripheral neurons and immune cells [14]. Difelikefalin is approved by regulatory entities including the US Food and Drug Administration (FDA) [16] and the European Medicines Agency [17] for the treatment of moderate-to-severe pruritus in patients undergoing HD [15, 18]. There is no evidence of addictive potential for difelikefalin, and the FDA has not labelled difelikefalin as a scheduled compound [14, 19, 20]. A safety analysis from four Phase 3 clinical studies showed that difelikefalin had an acceptable safety profile; the majority of adverse events (AEs) reported were mild or moderate in severity, with data on long-term use up to 52 weeks [21].

Many patients cannot enrol in a clinical trial or live in a location where a particular medication is not yet available. Managed access programmes (MAPs), while strictly regulated, provide a route by which these patients can receive new and novel treatments. Typical criteria for MAPs include [22]:

- The drug is indicated for the treatment of a serious medical condition for which no acceptable approved treatment is available.
- The benefits to the patient are likely to outweigh the risks.
- The programme will not disrupt current or planned clinical trials.
- The main objective is the provision of treatment rather than the collection of data for regulatory submissions.

Although the primary objective of a MAP ought to be providing treatment rather than collecting data, data from these programmes can also provide much-needed information on the safety of treatments in real-world settings [23]. While guidelines around the gathering of data from MAPs vary both within and between regions, it is acknowledged that data collection from these programmes can provide a valuable contribution to the overall data available on specific treatments [23]. The inclusion of more diverse populations in MAPs than would typically be included in clinical trials may also enable a better understanding of the effect of treatment in the clinical setting [23]. This is particularly important in medically complex populations such as patients with end-stage kidney disease (ESKD), for whom safety is key [24] but is complicated by comorbidities (such as cardiovascular disease, diabetes, hypertension, and increased risk of infection [25–27]), a high treatment burden, and mortality [25]. In these patients, long-term follow-up and adherence to trial protocols may be complicated, but real-world evidence from MAPs could provide useful insights.

The difelikefalin MAP reported here was initiated in October 2021; it provided patients with ESKD, who could benefit from difelikefalin treatment, access to this first-in-class medication before it became commercially available. The MAP continued in each country until difelikefalin received approval and was commercially available. Here, we describe the safety data obtained from the MAP between October 2021 and 31 October 2024 and compare it with the available Phase 3 clinical trial data.

## MATERIALS AND METHODS

### Programme design and treatment

The MAP provided difelikefalin to patients in locations where the treatment was not yet commercially available; it was designed as a pharmacovigilance initiative to collect real-world safety data on the use of difelikefalin. Eligible patients undergoing HD with moderate-to-severe CKD-aP were included following an unsolicited request by their healthcare professional (HCP) and received 0.5 µg/kg difelikefalin by intravenous bolus as part of their in-centre HD treatment (at the end of each dialysis session). The MAP began in October 2021, and the data analysed here were collected on 31 October 2024. The MAP was available to patients in Australia, Austria, Belgium, France, Germany, Ireland, Italy, Luxembourg, Portugal, Spain, and Switzerland. Once difelikefalin became commercially available in a country, all patients there were withdrawn from the MAP. Patients could continue using difelikefalin once the country withdrew from the MAP; however, no further safety data were collected.

### Eligibility criteria

Adult patients (>18 years old) were included in the MAP if they had ESKD, were undergoing in-centre HD three times per week, and had a moderate-to-severe level of pruritus (≥4 on the Worst Itching Intensity Numeric Rating Scale [28–30]) assessed by HCPs as being both attributable to ESKD (that is, CKD-aP) and having a significant impact on quality of life (based on whether the patient self-categorized as B or C using the self-assessed disease severity questionnaire [31]). Use of concomitant anti-pruritic medications (e.g. antihistamines or gabapentinoids) was allowed.

Patients were excluded if they were pregnant or breastfeeding, were hypersensitive to any of the ingredients in the treatment, had a severe hepatic impairment or impaired blood–brain barrier, or were taking part in (or planning to take part in) a clinical study.

The MAP was approved by the health authorities in each participating country in accordance with local regulatory mechanisms and carried out per the principles of the Declaration of Helsinki. Owing to the nature of this study, no central ethics committee approval was required for the collection of safety data from the MAP. All patients gave written informed consent prior to inclusion.

### Data collection

Data collection primarily focussed on safety, and the collection of data on baseline characteristics such as age and gender were not mandatory. HCPs reported AEs to the Local Drug Safety Responsible as soon as possible upon learning of the event. The Local Drug Safety Responsible then forwarded the cases to the Global Drug Safety (GDS) team, which was responsible for assessing the safety of difelikefalin and reviewing all investigator-completed safety reports over the course of the MAP. The GDS team then categorized events using pre-approved definitions for AEs, serious AEs (SAEs) [32], and special situations (including, but not limited to, drug ineffective, expired product administered, off-label use, or product quality issue) (Table 1), as well as assessing and determining causality (Table 1). Additional information about the safety events, such as severity, context, outcome, etc., was also collected and forwarded to the GDS team to assist in their full assessment. Time on treatment was estimated based on the dates of the first and last shipment of difelikefalin for each patient. Each shipment ensured treatment for 30 days, therefore, it is possible that the patient used less than the amount of difelikefalin provided.

**Table 1:** Definitions used in safety analysis [32, 36].

Term		Definition
Adverse event (AE)		Any untoward medical occurrence in a patient or clinical investigation subject who was administered a pharmaceutical product and that does not necessarily have to have a causal relationship with this treatment
		An AE can therefore be any unfavourable and unintended sign (including an abnormal laboratory finding), symptom, or disease temporally associated with the use of a medicinal product, whether or not related to the medicinal product
Serious adverse event (SAE)		An SAE is any untoward medical occurrence that at any dose: • Results in death • Is life-threatening • Requires inpatient hospitalization or prolongation of existing hospitalization • Results in persistent or significant disability/incapacity • Is a congenital anomaly/birth defect • Medically important event
Special situation		• Off-label use • Medication abuse • Medication error (this includes medication errors without an associated AE as well as intercepted medication errors ‘near-misses’) • Medication misuse • Medication overdose • Occupational exposure • Drug interaction • Unexpected therapeutic or clinical benefit from product use • Lack of efficacy • Pregnancy or lactation exposure with the product
Causality^a^	Certain	The event meets ≥1 of the following criteria: • Event or laboratory test abnormality, with plausible time relationship to drug administration • Cannot be explained by disease or other drugs • Response to withdrawal plausible (pharmacologically, pathologically) • Event definitive pharmacologically or phenomenologically (i.e. an objective and specific medical disorder or a recognized pharmacological phenomenon) • Rechallenge satisfactory, if necessary
	Probable	The event meets ≥1 of the following criteria: • Event or laboratory test abnormality, with reasonable time relationship to drug administration • Unlikely to be attributed to disease or other drugs • Response to withdrawal clinically reasonable • Rechallenge not required
	Possible	The event meets ≥1 of the following criteria: • Event or laboratory test abnormality, with reasonable time relationship to drug administration • Could also be explained by disease or other drugs • Information on drug withdrawal may be lacking or unclear
	Unlikely	The event meets ≥1 of the following criteria: • Event or laboratory test abnormality, with a time to drug administration that makes a relationship improbable (but not impossible) • Disease or other drugs provide plausible explanations
	Not related	The event meets the following criterion: • Event or laboratory test abnormality that is clearly related to circumstances not connected with the drug administration
Outcome	Recovered/resolved	The individual has recovered completely and has returned to the previous health status with no sequelae
	Recovered/resolving	The individual is in the process of recovering and has not yet returned to the previous health status
	Recovered/resolved with sequelae	Some permanent impairment to the health status has resulted after the event was resolved; the individual does not achieve previous health status
	Not recovered/not resolved	The individual continues in the same state that they were when the event was experienced
	Fatal	AE results in death of the subject
	Unknown	At the time of reporting, there is no information on the status of the individual after the event was experienced

a All points should be reasonably complied with.

## RESULTS

### Patient summary

There were 438 patients included in the MAP, who were provided with a median of 115 (min–max: 30–1035) days of treatment with difelikefalin. During the MAP safety collection period spanning 3 years, reported here, an estimated 95 628 cumulative days of difelikefalin treatment (262 years) was provided to the participating patients.

There were 319 patients who withdrew from the programme. Of the 11 countries initially included in the MAP, only three—Belgium, Italy, and Spain—were active in the MAP at the time of data collection (31 October 2024), and 119 patients in these countries were still undergoing treatment.

Of the patients for whom baseline data were available, the mean ± standard deviation age was 70.0 ± 13.7 years, and 67.1% of patients were male (Table 2).

**Table 2:** Baseline characteristics for patients experiencing AEs.

Characteristic	Patients experiencing AEs (*n* = 167)
Age, years	
*n* (missing)	106 (61)
Mean (SD)	70.1 (13.7)
Sex, patients (%)^a^	
*n* (missing)	161 (6)
Male	67.1%
Female	32.9%
Weight, kg	
*n* (missing)	89 (78)
Mean (SD)	75.1 (19.2)
Height, cm	
*n* (missing)	56 (111)
Mean (SD)	168.8 (7.2)
Country	
*n* (missing)	167 (0)
Australia	7.2%
Austria	2.4%
Belgium	37.7%
France	3.0%
Germany	10.2%
Ireland	0.6%
Italy	25.7%
Netherlands	0.6%
Spain	8.4%
Switzerland	4.2%

Collection of age, sex, weight and height were not mandatory.

^a^Percentage of patients for whom sex was recorded (*n* = 161).

SD, standard deviation.

### Safety events

At the data collection date for this analysis, 167 patients (38.1%) had experienced a total of 458 AEs. Of these, 246 (53.7%) were serious, 152 (33.2%) were non-serious, and 60 (13.1%) were special situations.

### Serious adverse events

There were 246 SAEs experienced by 87 patients (19.9%) (Table 3). The most common system organ classes (SOCs) for these SAEs were infections and infestations (14.6% of events), cardiac disorders (13.8% of events), surgical and medical procedures (12.2% of events), and general disorders and administration site conditions (12.2% of events).

**Table 3:** Serious adverse events.

	Events, *n* (%)
SAEs	246 (100.0)
SAEs by outcome	
Recovered/resolved	100 (40.1)
Recovered/resolving	14 (5.7)
Recovered/resolved with sequelae	4 (1.6)
Not recovered/not resolved	18 (7.3)
Unknown	47 (19.1)
Fatal	63 (25.6)
All SAEs by causality	
Probable	0 (0.0)
Possible	25 (10.2)
Unlikely	76 (30.9)
Not related	143 (58.1)
Not assessable^a^	2 (0.8)
Fatal SAEs by causality	
Probable	0 (0.0)
Possible	0 (0.0)
Unlikely	27 (42.9)
Not related	36 (57.1)
Not assessable	0 (0.0)
Blank	0 (0.0)
Most common SAEs (>1% of cases) by SOC	
Cardiac disorders	34 (13.8)
Infections and infestations	36 (14.6)
Surgical and medical procedures	30 (12.2)
General disorders and administration site conditions	30 (12.2)
Nervous system disorders	17 (6.9)
Respiratory, thoracic and mediastinal disorders	13 (5.3)
Injury, poisoning and procedural complications	13 (5.3)
Psychiatric disorders	12 (4.9)
Gastrointestinal disorders	11 (4.5)
Vascular disorders	11 (4.5)
Metabolism and nutrition disorders	10 (4.1)
Skin and subcutaneous tissue disorders	4 (1.6)
Hepatobiliary disorders	7 (2.8)
Eye disorders	4 (1.6)
Investigations	4 (1.6)
Musculoskeletal and connective tissue disorders	4 (1.6)
Neoplasms benign, malignant and unspecified (incl. cysts and polyps)	4 (1.6)
Most common SAEs (>1% of cases) by PT	
Pneumonia	10 (3.9)
Sepsis	9 (3.5)
Death	8 (3.1)
Confusional state	6 (2.3)
Drug reaction with eosinophilia and systemic symptoms	6 (2.3)
Hyperkalaemia	6 (2.3)
Angioplasty	5 (1.9)
Coronary angioplasty	5 (1.9)
Renal transplant	5 (1.9)
Angina pectoris	4 (1.5)
Cholangitis	4 (1.5)
Coronary arterial stent insertion	4 (1.5)
Pyrexia	4 (1.5)
Acute myocardial infarction	3 (1.2)
Arteriovenous fistula site complication	3 (1.2)
Atrioventricular block complete	3 (1.2)
Bradycardia	3 (1.2)
Cardiac arrest	3 (1.2)
Cardiac failure	3 (1.2)
Cardiac pacemaker insertion	3 (1.2)
Cardiogenic shock	3 (1.2)
Disorientation	3 (1.2)
Fall	3 (1.2)
Hypoglycaemia	3 (1.2)
Hypotension	3 (1.2)
Multiple organ dysfunction syndrome	3 (1.2)
Myocardial infarction	3 (1.2)
Nausea	3 (1.2)
Pulmonary embolism	3 (1.2)
Somnolence	3 (1.2)

^a^There were two cases of drug ineffective. Per convention, the causality of special situations is considered ‘not assessable’.

SAEs, SOCs and PTs all defined by MedDRA classification.

MedDRA, Medical Dictionary for Regulatory Activities Terminology.

**Figure 1:**
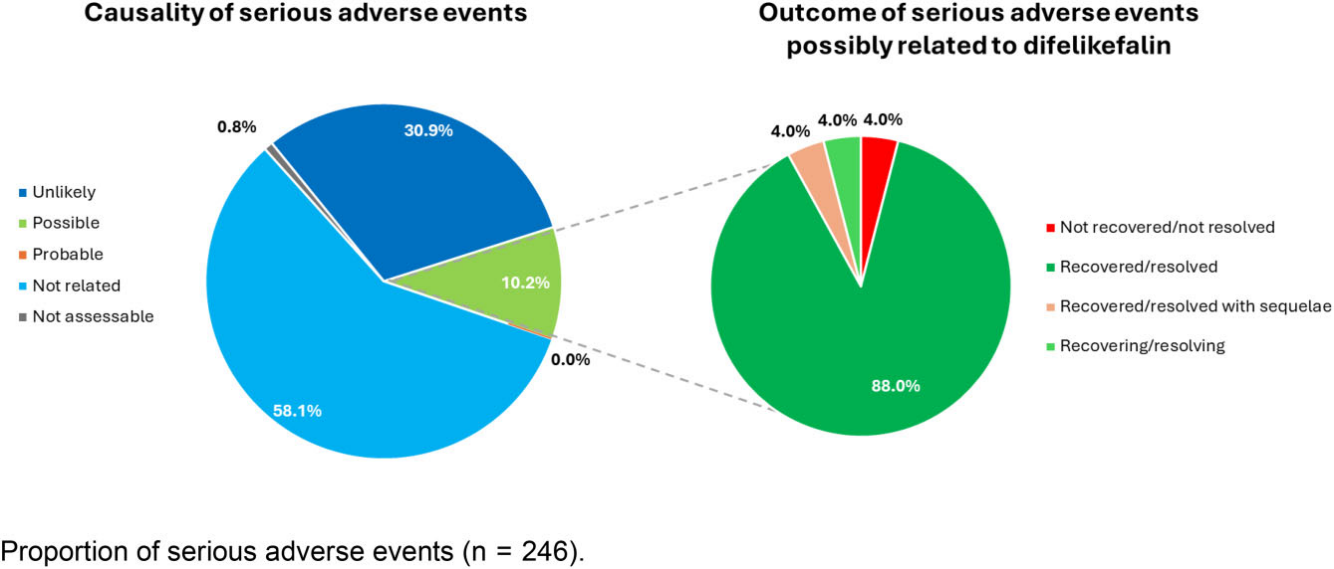
SAEs (*n* = 246)—causality with difelikefalin and outcomes of SAEs possibly related to difelikefalin.

Outcomes were recorded for 199 SAEs and causality was recorded for 246 SAEs. In 89.0% of cases, SAEs were considered unrelated or unlikely to be related to difelikefalin by the GDS team; causality was not assessable in 0.8% of cases, while 10.2% of SAEs were considered possibly related to difelikefalin, and no SAEs were considered probably related to difelikefalin (Fig. 1). Of the SAEs for which an outcome was known, 88.0% of those that were considered possibly related to difelikefalin were resolved or recovered, while 4.0% were recovering/resolving, 4.0% were recovered/resolved with sequelae, and 4.0% were not recovered/not resolved (Fig. 1, Table 4). Of those considered to be related to difelikefalin that were resolved, 1 event in 1 patient was resolved following hospitalization and treatment, and 6 events in 3 patients were resolved following discontinuation of difelikefalin; there are no details of remedial actions for 15 events in 6 patients.

**Table 4:** SAEs considered possibly related to difelikefalin.

	Events, *n* (%)
SAEs possibly related to difelikefalin	25 (100.0)
SAEs possibly related to difelikefalin by outcome	
Recovered/resolved	22 (88.0)
Recovered/resolving	1 (4.0)
Recovered/resolved with sequelae	1 (4.0)
Not recovered/not resolved	1 (4.0)
SAEs possibly related to difelikefalin by SOC	
Nervous system disorders	6 (24.0)
Gastrointestinal disorders	5 (20.0)
Metabolism and nutrition disorders	4 (16.0)
Psychiatric disorders	4 (16.0)
Ear and labyrinth disorders	1 (4.0)
Skin and subcutaneous tissue disorders	1 (4.0)
General disorders and administration site conditions	1 (4.0)
Injury, poisoning and procedural complications	1 (4.0)
Investigations	1 (4.0)
Vascular disorders	1 (4.0)
SAEs possibly related to difelikefalin by PT	
Hyperkalaemia	4 (16.0)
Confusional state	3 (12.0)
Nausea	3 (12.0)
Dizziness	2 (8.0)
Loss of consciousness	2 (8.0)
Vomiting	2 (8.0)
Disorientation	1 (4.0)
Drug reaction with eosinophilia and systemic symptoms	1 (4.0)
Electrocardiogram QT prolonged	1 (4.0)
Fall	1 (4.0)
Fatigue	1 (4.0)
Hypotension	1 (4.0)
Somnolence	1 (4.0)
Syncope	1 (4.0)
Vertigo	1 (4.0)

SAEs, SOCs and PTs all defined by MedDRA classification.

MedDRA, Medical Dictionary for Regulatory Activities Terminology.

### Fatal adverse events

**Figure 2:**
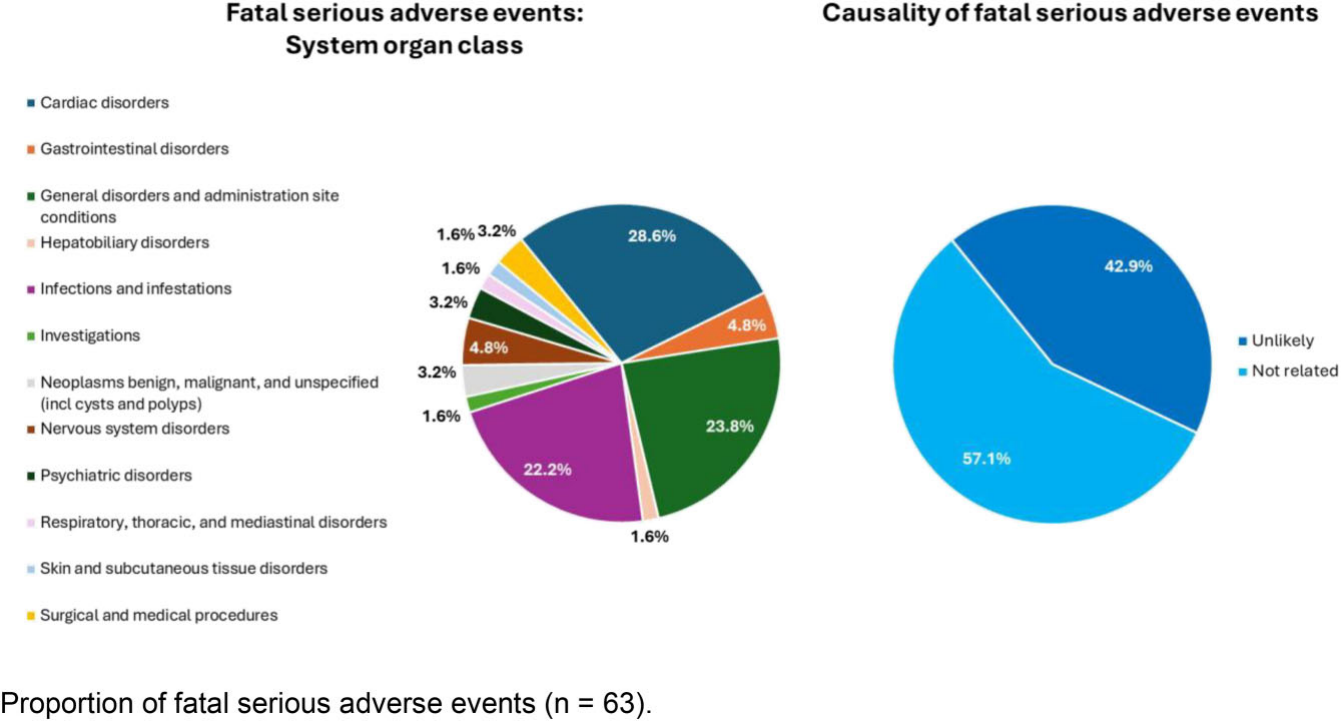
Fatal SAEs (*n* = 63)—system organ class and causality with difelikefalin.

There were 63 fatal events, resulting in the deaths of 43 patients (9.8%; fatal SAEs were not mutually exclusive, meaning multiple fatal SAEs were reported for some patients) during the MAP data collection period (Table 3). By SOC level (Fig. 2), deaths primarily resulted from cardiac disorders (28.6% of events, e.g. myocardial infarction, cardiac failure), general disorders and administration site conditions (23.8% of events, e.g. general physical health deterioration, multiple organ dysfunction syndrome), and infections and infestations (22.2% of events, e.g. sepsis, pneumonia). None of the fatal events were considered related to difelikefalin by the GDS team; 57.1% of events were considered not related, and 42.9% of events were unlikely to be related to difelikefalin treatment (Fig. 2).

### Non-fatal serious adverse events

There were 183 non-fatal SAEs experienced by 54 patients (12.3%). The most frequently occurring SOCs for the non-fatal SAEs were surgical and medical procedures (15.3%), infections and infestations (12.0%), cardiac disorders (8.7%), and general disorders and administration site conditions (8.2%).

The 16 events classified as under the SOC of cardiac disorders occurred in eight patients (1.8%) and included acute myocardial infarction, angina pectoris, atrial fibrillation, atrioventricular block complete, bradycardia, cardiac failure, cardiogenic shock, cardio-respiratory arrest, coronary artery disease, and left ventricular dysfunction. None of these events was considered by the GDS team to be related to difelikefalin.

### Non-serious adverse events

There were 152 non-serious AEs experienced by 85 patients (19.4%) during the MAP (Table 5). The most frequently occurring SOC for the non-serious AEs reported were general disorders and administration site conditions (19.7% of events), nervous system disorders (25.0% of events), and gastrointestinal disorders (15.8% of events). Among the non-serious AEs with an incidence of at least 2%, the ones with higher incidence by preferred term (PT) were somnolence (7.9% of events), dizziness (6.6% of events), pruritus (6.6% of events) and diarrhoea (5.9% of events).

**Table 5:** Non-serious AEs.

	Events, *n* (%)
Non-serious AEs	152 (100.0)
Non-serious AEs by outcome	
Recovered/resolved	75 (49.3)
Recovered/resolving	10 (6.6)
Recovered/resolved with sequelae	0 (0.0)
Not recovered/not resolved	7 (4.6)
Unknown	60 (39.5)
Fatal	0 (0.0)
Non-serious AEs by causality	
Probable	7 (4.6)
Possible	90 (59.2)
Unlikely	25 (16.4)
Not related	25 (16.4)
Not assessable	5 (3.3)
Most common non-serious AEs (>1% of cases) by SOC	
Nervous system disorders	38 (25.0)
General disorders and administration site conditions	30 (19.7)
Gastrointestinal disorders	24 (15.8)
Skin and subcutaneous tissue disorders	18 (11.8)
Psychiatric disorders	16 (10.5)
Infections and infestations	8 (5.3)
Musculoskeletal and connective tissue disorders	7 (4.6)
Injury, poisoning, and procedural complications	6 (3.9)
Respiratory, thoracic, and mediastinal disorders	3 (2.0)
Most common non-serious AEs (>1% of cases) by PT^a^	
Somnolence	12 (7.9)
Dizziness	10 (6.6)
Pruritus	10 (6.6)
Diarrhoea	9 (5.9)
Disease progression	7 (4.6)
Nausea	6 (3.9)
Adverse event	5 (3.3)
Headache	5 (3.3)
Condition aggravated	4 (2.6)
Constipation	4 (2.6)
Disorientation	4 (2.6)
Fall	4 (2.6)
Confusional state	3 (2.0)
Insomnia	3 (2.0)
Mental status changes	3 (2.0)
Muscular weakness	3 (2.0)
Myalgia	3 (2.0)
Cognitive disorder	2 (1.3)
COVID-19	2 (1.3)
Eczema	2 (1.3)
Fatigue	2 (1.3)
Feeling abnormal	2 (1.3)
Paraesthesia	2 (1.3)
Sedation	2 (1.3)
Unevaluable event	2 (1.3)
Vomiting	2 (1.3)

^a^There were 43 cases of drug ineffective, these are listed as special situations and are not listed here.

AEs, SOCs and PTs all defined by MedDRA classification.

MedDRA, Medical Dictionary for Regulatory Activities Terminology.

The majority of the non-serious AEs reported (63.8% of events) were considered possibly (59.2% of events) or probably (4.6% of events) related to difelikefalin by the GDS team, while the remaining 36.2% of non-serious AEs were either not related or unlikely to be related to difelikefalin (32.9% of events), or were not assessable as their PTs were no AEs or unevaluable event (3.3% of events).

Of the 59 non-serious AEs possibly or probably related to difelikefalin for which the outcome is known, 86.4% (51 events) were recovered/resolved by the data collection date.

### Special situations

There were 60 special situations recorded for 56 patients (12.8%; Table 6). The most reported special situations were lack of drug effect/drug ineffective (68.3% of events), off-label use (6.7% of events), product quality issue (5.0% of events), and therapy non-responder (5.0% of events). When similar PTs (drug ineffective, therapeutic product effect decreased, and therapy non-responder) were combined, lack of therapeutic effect accounted for 45 (75.0%) of special situations. Overall, 16 patients (3.7%) had <84 estimated days (12 weeks) of difelikefalin treatment before discontinuing. In five patients (1.1%), drug ineffective was reported alongside pruritus as an AE. For 27 patients (6.2%), AEs relating to the lack of therapeutic effect (drug ineffective, therapeutic product effect decreased or therapy non-responder) were the only reported AEs.

**Table 6:** Special situations.

	Events, *n* (%)
All special situations	60 (100.0)
Drug ineffective	41 (68.3)
Off-label use	4 (6.7)
Product quality issue	3 (5.0)
Therapy non-responder	3 (5.0)
Product storage error	2 (3.3)
Expired product administered	1 (1.7)
Inappropriate schedule of product administration	1 (1.7)
Product complaint	1 (1.7)
Product dose omission issue	1 (1.7)
Product prescribing issue	1 (1.7)
Therapeutic product effect decreased	1 (1.7)
Therapy cessation	1 (1.7)

Two special situations in two patients were considered serious by the GDS team. In one patient, symptoms of itch increased significantly following difelikefalin initiation and sedatives and antihistamines were initiated. In the second, the patient did not experience any benefits or report any improvement with treatment. In both cases, the patients discontinued difelikefalin. All other special situations (96.8%) were considered non-serious.

## DISCUSSION

No new safety signals in the use of difelikefalin were identified in this analysis, in which difelikefalin demonstrated a safety profile similar to that observed in Phase 3 clinical trials.

In a pooled analysis of the KALM-1, KALM-2, CLIN3101, and CLIN3105 clinical trials, the incidence of treatment-emergent AEs was 71.2% in the difelikefalin group versus 65.3% in the placebo group, with the majority of events reported as mild or moderate in severity [21]. Few participants receiving difelikefalin (6.8%) discontinued due to treatment-emergent AEs, and no deaths were considered to be related to the study drug [21].

Of the SAEs in this assessment, the SOCs occurring most frequently—such as cardiac conditions, infections and infestations, surgical and medical procedures, and general disorders and administration site conditions—are common in older adults with ESKD receiving HD [25–27]. In this analysis, few SAEs were considered possibly or probably related to difelikefalin by the GDS team, and the majority of those considered related to difelikefalin were resolved. Similarly, most SAEs resulting in death occurred owing to complications that are common in this patient population (cardiac disorders, general disorders and administration site conditions, and infections and infestations [25–27]), and no fatal SAEs or cardiac disorder SAEs were considered to be possibly or probably related to difelikefalin [21].

Similarly, the non-serious AEs reported in the MAP were common in this patient population as a result of their comorbid conditions or were related to those listed in the prescribing information for difelikefalin [15]. The majority of non-serious AEs considered to be related to difelikefalin by the GDS team were resolved during the MAP.

The results of this MAP are aligned and consistent with those of Phase 3 difelikefalin clinical trials, in which the most common AEs in patients receiving difelikefalin were diarrhoea, dizziness, nausea, gait disturbances (including falls), hyperkalaemia, headache, somnolence, and mental state changes [21]. These AEs were mostly mild or moderate (≥65% of events), with few leading to study drug discontinuation [21]. In a sub-analysis of the KALM-1 and KALM-2 clinical trials, the common AEs of diarrhoea, dizziness, and nausea tended to occur within the first 40 days of treatment and had a duration of up to 3 days, which suggested that AEs typically resolved, while difelikefalin treatment was ongoing [33]. Similar results were observed in a 15-patient MAP conducted in Germany, in which the most common AEs were dizziness and headache, and most were mild in severity [34].

While a number of patients included in this analysis discontinued difelikefalin owing to lack of effectiveness, half of the patients who discontinued for this reason (and for whom time on treatment was recorded) received <12 estimated weeks of treatment. This is noteworthy, as in Phase 3 clinical trials, 12 weeks was noted as the length of time for which difelikefalin should be taken before discontinuing owing to lack of efficacy.

This analysis provides real-world evidence for the safety profile of difelikefalin in the treatment of CKD-aP and demonstrates consistency with reports from the Phase 3 clinical trials [21]. This is important because real-world populations are heterogeneous, and the evidence provided from this MAP gives an insight into the safety of treatments in a less controlled setting [35], and is particularly important for chronic and complex conditions, such as CKD-aP.

A key strength of this analysis is its use of a real-world population, in which the controlled settings of a clinical trial cannot be replicated. Despite the uncontrolled environment and the inclusion of a population with a range of comorbidities, no new safety signals were detected during this MAP. This may provide reassurance to clinicians that difelikefalin has an acceptable safety profile for their patients. Another strength of this study is the long time period (3 years) over which the safety of difelikefalin was monitored.

This study has several limitations that should be considered when interpreting the results. As part of a MAP, data were collected through routine pharmacovigilance reporting in a real-world clinical setting. Consequently, no efficacy data were recorded, and the rigor of safety data collection did not match that of controlled clinical trials. Baseline demographic and clinical characteristics were sometimes incomplete, limiting the feasibility of subgroup analyses.

Safety data were also not captured consistently for all patients. In some cases, key information, such as the dates of difelikefalin initiation and discontinuation, as well as the timing of adverse event onset and resolution, was missing. This limitation, inherent to the real-world design of the study, precluded analysis of the temporal relationship between treatment initiation and the occurrence of adverse events, including potential differences by event type. We would recommend that future studies systematically collect these data in order to conduct more detailed analyses, such as how adverse event reporting changes over time. Furthermore, time on treatment was estimated based on the dates of the first and last shipment of difelikefalin for each patient. As each shipment ensured treatment for 30 days, it is possible that the patients used less difelikefalin than estimated.

Notably, safety information collected during the MAP relied on patient self-reporting; therefore, it is possible that reporting bias may have been introduced, or that some AEs may not have been reported. As with the Phase 3 clinical trials, most patients continued previous anti-pruritic medication while receiving difelikefalin; the impact of these concomitant medications on the safety profile has not been explored. Finally, efficacy data, which may have aided interpretation of AEs attributed to drug inefficacy, were not recorded.

## CONCLUSION

No new safety signals in the use of difelikefalin were identified in this analysis of real-world data from a large MAP in Europe and Australia, which provided difelikefalin to adult patients with moderate-to-severe CKD-aP and for whom safety data were collected over a period of 3 years.

This analysis provides additional evidence for the consistent safety profile of difelikefalin, confirms the safety data from the Phase 3 clinical trial programme in a real-world setting, and may provide reassurance to clinicians regarding the safety of difelikefalin. Considering the available Phase 3 clinical efficacy data, it can be concluded that difelikefalin has a favourable safety profile for the treatment of CKD-aP in patients receiving HD.

## Data Availability

The data underlying this article will be shared on reasonable request to the corresponding author.

## References

[1] Jha CM, Dastoor HD, Gopalakrishnan N et al. Obstacles to early diagnosis and treatment of pruritus in patients with chronic kidney disease: current perspectives. Int J Nephrol Renovasc Dis 2022;15:335–52. 10.2147/IJNRD.S294147 36510564 PMC9739055

[2] Lipman ZM, Paramasivam V, Yosipovitch G et al. Clinical management of chronic kidney disease-associated pruritus: current treatment options and future approaches. Clin Kidney J 2021;14:i16–22. 10.1093/ckj/sfab167 34987779 PMC8702820

[3] Areste N, Sanchez-Alvarez JE, Prieto-Velasco M et al. Prevalence and severity of pruritus in Spanish patients with chronic kidney disease and impact on quality of life: a cross-sectional study. Clin Kidney J 2023;16:1035–7. 10.1093/ckj/sfac246 37260996 PMC10229293

[4] Lanot A, Bataille S, Rostoker G et al. Moderate-to-severe pruritus in untreated or non-responsive hemodialysis patients: results of the French prospective multicenter observational study Pruripreva. Clin Kidney J 2023;16:1102–12. 10.1093/ckj/sfad032 37398693 PMC10310516

[5] Rayner HC, Larkina M, Wang M et al. International comparisons of prevalence, awareness, and treatment of pruritus in people on hemodialysis. Clin J Am Soc Nephrol 2017;12:2000–7. 10.2215/CJN.03280317 28923831 PMC5718267

[6] Agarwal P, Garg V, Karagaiah P et al. Chronic kidney disease-associated pruritus. Toxins 2021;13:527. 10.3390/toxins13080527 34437400 PMC8402524

[7] Ramakrishnan K, Bond TC, Claxton A et al. Clinical characteristics and outcomes of end-stage renal disease patients with self-reported pruritus symptoms. Int J Nephrol Renovasc Dis 2013;7:1–12. 24379689 10.2147/IJNRD.S52985 PMC3872274

[8] Shirazian S, Aina O, Park Y et al. Chronic kidney disease-associated pruritus: impact on quality of life and current management challenges. Int J Nephrol Renovasc Dis 2017;10:11–26. 10.2147/IJNRD.S108045 28176969 PMC5271405

[9] Weiss M, Mettang T, Tschulena U et al. Health-related quality of life in haemodialysis patients suffering from chronic itch: results from GEHIS (German Epidemiology Haemodialysis Itch Study). Qual Life Res 2016;25:3097–106. 10.1007/s11136-016-1340-4 27307011

[10] Kini SP, DeLong LK, Veledar E et al. The impact of pruritus on quality of life: the skin equivalent of pain. Arch Dermatol 2011;147:1153–6. 10.1001/archdermatol.2011.178 21680760

[11] Kimata N, Fuller DS, Saito A et al. Pruritus in hemodialysis patients: results from the Japanese Dialysis Outcomes and Practice Patterns Study (JDOPPS). Hemodial Int 2014;18:657–67. 10.1111/hdi.12158 24766224

[12] Sukul N, Karaboyas A, Csomor PA et al. Self-reported pruritus and clinical, dialysis-related, and patient-reported outcomes in hemodialysis patients. Kidney Med 2021;3:42–53.e1. 10.1016/j.xkme.2020.08.011 33604539 PMC7873756

[13] Thompson J, Kammerer J, Boshears T et al. Chronic kidney disease-associated pruritus burden: a patient survey study. Kidney Med 2024;6:100900. 10.1016/j.xkme.2024.100900 39822582 PMC11738028

[14] Agarwal R, Burton J, Gallieni M et al. Alleviating symptoms in patients undergoing long-term hemodialysis: a focus on chronic kidney disease-associated pruritus. Clin Kidney J 2023;16:30–40. 10.1093/ckj/sfac187 36726430 PMC9871858

[15] Vifor CSL. Kapruvia SmPC. 2022. Available from: https://newsroom.csl.com/2021-08-24-Vifor-Pharma-and-Cara-Therapeutics-announce-U-S-FDA-approval-of-KORSUVA-TM-injection-for-the-treatment-of-moderate-to-severe-pruritus-in-hemodialysis-patients (date last accessed, 25 April 2025).

[16] US Food & Drug Administration . NDA Approval: Difelikefalin. 2021. Available from: https://www.accessdata.fda.gov/drugsatfda_docs/appletter/2021/214916Orig1s000ltr.pdf (date last accessed, 3 April 2025).

[17] European Medicines Agency . Kapruvia (Difelikefalin). 2022. Available from: https://www.ema.europa.eu/en/medicines/human/EPAR/kapruvia#authorisation-details (date last accessed, 3 April 2025).

[18] CSL Vifor Pharma . Vifor Pharma and Cara Therapeutics Announce U.S. FDA Approval of Korsuva™ Injection for the Treatment of Moderate to Severe Pruritus in Hemodialysis Patients 2021. Available from: https://newsroom.csl.com/2021-08-24-Vifor-Pharma-and-Cara-Therapeutics-announce-U-S-FDA-approval-of-KORSUVA-TM-injection-for-the-treatment-of-moderate-to-severe-pruritus-in-hemodialysis-patients#:∼:text=St.%20Gallen%2C%20Switzerland%2C%20and%20Stamford%2C%20Conn%2C%2024%20August,with%20chronic%20kidney%20disease%20in%20adults%20undergoing%20hemodialysis (date last accessed, 25 April 2025).

[19] Shram MJ, Spencer RH, Qian J et al. Evaluation of the abuse potential of difelikefalin, a selective kappa-opioid receptor agonist, in recreational polydrug users. Clin Transl Sci 2022;15:535–47. 10.1111/cts.13173 34708917 PMC8841457

[20] Spencer RH, Munera C, Shram MJ et al. Assessment of the physical dependence potential of difelikefalin: randomized placebo-controlled study in patients receiving hemodialysis. Clin Transl Sci 2023;16:1559–68. 10.1111/cts.13538 37128642 PMC10499405

[21] Fishbane S, Wen W, Munera C et al. Safety and tolerability of difelikefalin for the treatment of moderate to severe pruritus in hemodialysis patients: pooled analysis from the phase 3 clinical trial program. Kidney Med 2022;4:100513. 10.1016/j.xkme.2022.100513 36039153 PMC9418597

[22] Wasserstrom D. Supplementing Clinical Development with a Managed Access Program. 2017. Available from: https://www.clinigengroup.com/insight/insights/2017/supplementing-clinical-development-with-a-managed-access-program/ (date last accessed, 25 April 2025).

[23] Sarp S, Reichenbach R, Aliu P. An approach to data collection in compassionate use/managed access. Front Pharmacol 2022;13:1095860. 10.3389/fphar.2022.1095860 36605403 PMC9810195

[24] Whittaker CF, Miklich MA, Patel RS et al. Medication safety principles and practice in CKD. Clin J Am Soc Nephrol 2018;13:1738–46. 10.2215/CJN.00580118 29915131 PMC6237057

[25] MacRae C, Mercer SW, Guthrie B et al. Comorbidity in chronic kidney disease: a large cross-sectional study of prevalence in Scottish primary care. Br J Gen Pract 2021;71:e243–9. 10.3399/bjgp20X714125 33558333 PMC7888754

[26] Berman SJ, Johnson EW, Nakatsu C et al. Burden of infection in patients with end-stage renal disease requiring long-term dialysis. Clin Infect Dis 2004;39:1747–53. 10.1086/424516 15578394

[27] Cha J, Han D. Health-related quality of life based on comorbidities among patients with end-stage renal disease. Osong Public Health Res Perspect 2020;11:194–200. 10.24171/j.phrp.2020.11.4.08 32864310 PMC7442444

[28] Phan NQ, Blome C, Fritz F et al. Assessment of pruritus intensity: prospective study on validity and reliability of the visual analogue scale, numerical rating scale and verbal rating scale in 471 patients with chronic pruritus. Acta Derm Venerol 2012;92:502–7. 10.2340/00015555-1246 22170091

[29] Storck M, Sandmann S, Bruland P et al. Pruritus Intensity Scales across Europe: a prospective validation study. Acad Dermatol Venereol 2021;35:1176–85. 10.1111/jdv.17111 33411947

[30] Naegeli AN, Flood E, Tucker J et al. The Worst Itch Numeric Rating Scale for patients with moderate to severe plaque psoriasis or psoriatic arthritis. Int J Dermatol 2015;54:715–22. 10.1111/ijd.12645 25515935

[31] Mathur VS, Lindberg J, Germain M et al. A longitudinal study of uremic pruritus in hemodialysis patients. Clin J Am Soc Nephrol 2010;5:1410–9. 10.2215/CJN.00100110 20558560 PMC2924419

[32] European Medicines Agency . Note for Guidance on Clinical Safety Data Management: Definitions and Standards for Expedited Reporting (CPMP/ICH/377/95). 1995. Available from: https://www.ema.europa.eu/en/documents/scientific-guideline/international-conference-harmonisation-technical-requirements-registration-pharmaceuticals-human-use-topic-e-2-clinical-safety-data-management-definitions-and-standards-expedited-reporting-step_en.pdf (25 April 2025, date last accessed).

[33] Reddy JK, McCafferty K, Munera C et al. Characterization of Common Adverse Reactions Observed With Intravenous Difelikefalin for the Treatment of Chronic Kidney Disease–Associated Pruritus in Adults Undergoing Hemodialysis. American Society of Nephrology: Kidney Week, Pennsylvania, USA: 2023.

[34] Kraft L, Schanz M, Schricker S et al. The first real-world experience of IV difelikefalin to treat chronic kidney disease-associated pruritus in haemodialysis patients. Acad Dermatol Venereol 2023;37:e1059–61. 10.1111/jdv.19105 37016989

[35] Sherman RE, Anderson SA, Dal Pan GJ et al. Real-world evidence—what is it and what can it tell us? N Engl J Med 2016;375:2293–7. 10.1056/NEJMsb1609216 27959688

[36] Centre TUM . The Use of the WHO-UMC System for Standardised Case Causality Assessment. 2013. Available from: https://cdn.who.int/media/docs/default-source/medicines/pharmacovigilance/whocausality-assessment.pdf?sfvrsn=5d8130bb_2&download=true (date last accessed, 25 April 2025).

